# Using Bishop’s Card Reaching Task to Assess Hand Preference in 8- to 10-Year-Old Czech Children

**DOI:** 10.1371/journal.pone.0166337

**Published:** 2016-11-11

**Authors:** Martin Musalek, Sara Marie Scharoun, Pamela J. Bryden

**Affiliations:** 1 Faculty of Physical Education and Sport, Charles University, Prague, Czech Republic; 2 School of Physical and Health Education, Nipissing University, North Bay, Ontario, Canada; 3 Department of Kinesiology and Physical Education, Wilfrid Laurier University Waterloo, Ontario, Canada; University of Ottawa, CANADA

## Abstract

Hand preference is one of the most apparent functional asymmetry in humans. Under contralateral control, performance is more proficient with the preferred hand; however, the difference between the two hands is greater in right handers, considering left handers generally display less cerebral lateralization. One method of evaluating hand preference is Bishop’s card reaching task; however, information regarding validity and sensitivity with children in limited. This study assessed the relationship between Bishop’s card reaching task and five hand preference tasks in 8- to 10-year-old typically-developing children from the Czech Republic (*N* = 376). Structural equation modelling identified a one factor model as the most suitable, including Bishop’s card reaching task and three hand preference tasks (ringing, throwing, and rolling with dice). The factor validity (.89) and sensitivity of Bishop’s card reaching task (90% to 97%) provided a very good identification of hand preference. These results support the suitability of Bishop’s card reaching task as a separate test for determining hand preference in children. Accordingly, we suggest that the assessment of handedness, particularly in neurodevelopmental disorders where the proportion of right-handers and left-handers is disrupted (e.g., children with DCD or ADHD), should make use of Bishop’s card reaching task alongside other unimanual tasks.

## Introduction

Handedness represents the most apparent and studied functional asymmetry in humans [1]. Under contralateral control, the left hemisphere is responsible for right hand function, and the right hemisphere for left hand function [2, 3]. Our understanding of cerebral lateralization dates back to the work of Paul Broca, who observed the effects of left hemisphere lesions in the posterior part of the third frontal convolution in right handers. Broca proposed that cerebral control for speech was specific to one hemisphere, and mirrored an individual’s handedness [4, 5]. It has since been observed that the left hemisphere is stereotypically responsible for language and motor skills, and the right hemisphere is responsible for processing visuospatial information. Nevertheless, despite 87–96% of the population displaying left hemisphere lateralization for language, not all are right-handed. Approximately 60–73% of left-handers also fall under this distinction; whereas others display bilateral distribution across hemispheres, or right hemisphere lateralization [6, 7]. The aforementioned distributions have been confirmed using functional transcranial sonography [6, 7], repetitive transcranial magnetic stimulation [8] and functional magnetic resonance imaging (fMRI)[9].

Defined behaviourally in terms of *preference* (i.e., the preferential use of one of the hands) and *performance* (i.e., differentiating based on abilities in a particular task)[10], 90% of the population is right-handed and 10% is left-handed [11, 12]. This distribution has remained relatively consistent for approximately 5000 years [11]. Examining the relationship between preference and performance, it is commonly reported that performance is more proficient with the preferred hand [13]. However, the difference between the two hands is typically greater in right than left handers, considering left handers generally display less functional asymmetry than right handers [14, 15]. In one example, Jäncke et al. [16] have reported, with fMRI, that right handers require an increase in effort to perform with their left hand. As such, right handers display greater activation in the right hemisphere when using the left hand, than in the left hemisphere when using the right hand [16]. Other researchers have similarly identified greater activation in contralateral motor areas [17, 18].

Numerous behavioural tools are available for the evaluation of hand preference. Questionnaires are the most widely used, where participants record their response to a series of unimanual tasks. Questionnaires such as the Edinburgh Handedness Inventory [19], the Waterloo Handedness Questionnaire [20], and the Annett Handedness Questionnaire [13] are commonly used in the literature; however, this method of evaluating hand preference comes with certain limitations. More particularly, limitations include the subjective nature of responses [21] and the consideration that no single questionnaire was explicitly designed to assess children. Therefore, some authors suggest that performance-based measures of preference represent a more objective approach to evaluate hand preference, especially with children [22, 23].

One of these performance-based measures is the Quantification of Hand Preference (QHP) task [24], which assesses hand preference in reaching throughout regions of hemispace. Also assessed in reference to manual midline crossing, it has been suggested that the emergence of such behaviour reflects a shift from extracallosal to callosal control of interhemispheric communication [25] and is thus a prerequisite from developing a skilled preferred hand [26, 27, 28]. The original version of the QHP was limited to one task (i.e., Bishop’s card reaching task). Here, seven coloured cards were placed at 30-degree intervals within hemispace. Participants were seated at a table with the task in front of them, and asked to grasp a card of a certain colour and place it into a box located at the midline. The hand used to pick up the card in each region of hemispace was recorded [24]. The QHP displays high homogeneity and test-retest reliability (.78 - .80) [29]. Furthermore, Bishop et al. [24] were able to successfully identify right handers based on degree of hand preference [1]. A reflection of how strongly a person prefers one hand, degree of hand preference is a behavioural reflection of cerebral lateralization for handedness. This has been reported in several studies assessing both structural and functional cortical organization [30, 31, 32]. For example, using resting-state fMRI, Pool et al. [32] identified stronger interhemispheric functional connectivity in right handers. As such, the authors suggested functional connectivity between left primary motor cortex and right dorsolateral premotor cortex may be used as an indicator of handedness [32]. Extending Bishop et al.’s [24] findings, Calvert and Bishop [32] repeated the QHP with right- and left-handers to investigate whether left-handers mirror right-handers, or display less cerebral lateralization. Two additional tasks were added to the QHP (pointing to a letter and placing/posting a marble) to examine the role that skill played in the degree of preference. Overall, the QHP was sensitive to degree of hand preference both within and between groups of left- and right-handers; however, the pointing and placing tasks proved to be more effective assessments [24, 32]. This finding clearly outlines the need to consider the difficulty of the task when assessing hand preference [32, 33].

More recent investigations have examined the QHP task from a developmental perspective. It is generally reported that the shift from immature to mature motor control strategies at approximately age 10 to 12 may reflect maturation of the corpus callosum [9, 34, 35, 36, 37]. Failure to cross the midline by age 3 to 4 has thus been identified as a marker for potential perceptual-motor difficulties later in life [38]. With respect to the QHP, some [39, 40] have limited inquiries to the card-reaching task, and others [41] have examined all three components (i.e., reaching, pointing and posting). Overall, findings reveal younger children (i.e., 3- to 5-year-olds) have weaker cerebral lateralization, and hand preference tendencies; therefore, are less likely to cross the midline in comparison to older children (i.e., 7- to 12-year-olds) who are strongly lateralized, and thus reliant on their preferred hand. Adults, in comparison, will reach into ipsilateral space with either the preferred or non-preferred hand reflecting acquired motor skills, which decrease complexity [23].

Whereas the aforementioned studies utilized the QHP to investigate hand preference in manual midline crossing, other studies have modified the task to include other objects. In one example, Bryden et al. [42] placed everyday tools (e.g., pen, toothbrush, hammer, paint brush, spoon) at 45-degree angles in peripersonal space. Adult participants were first asked to lift an object and demonstrate the action as if it were a tool. Hand preference was stronger in the tasks that involved demonstration [42]. These findings have been replicated with both adults and children [43]. Overall, results of these studies exemplify the link between hand preference and manual midline crossing over the course of development, where children in the 7- to 10-year-old age range cross the midline significantly more than other age groups (both younger and older) [23, 39, 40, 43].

In summary, the previous literature suggests that the QHP is a suitable measure of human handedness in children and adults. Nevertheless, the quality of the card reaching task (i.e., validity) has not been verified in children. Differences in performance have been shown to reflect changes in cerebral lateralization with age, and midline crossing has been used to infer maturation of the corpus callosum. As such, it is of utmost importance that behavioural measurements tools are both valid and reliable. Therefore, the aim of this study was to examine the factor validity of Bishop’s card reaching task. Furthermore, a secondary aim of this study was to assess discriminant and convergent validity between the task, and a selection of five standard assessments of hand preference in 8- to 10-year-old children. It was hypothesized that the task would be identified as a valid assessment for children and be highly correlated (r > .80) with other measures of handedness, considering children in this age range are strongly lateralized and thus display consistent hand preference tendencies. The research was focused specifically on describing the defined phenomenon and therefore the study was observational in nature [44].

A threshold of r > .80 was selected based on several considerations, including work by Kline [45], descibing convergent validity as a correlation at least of moderate strength. There are several guidelines which can be used to identify a sufficiently high correlation. For example, Cohen [46] and Hendl [47] identify a large effect as >.50 or >.70. Brown [48] discussed sufficient convergent validity in the range of .676 to .749. With respect to reliability, when observing the correlation between the card-reaching task and other validated measures of handedness, this can be expressed similar to internal consistency, where the generally acceptable level is recommended to be greater than .80 [49, 50].

## Materials and Methods

### Participants

The current study included 376 children (184 boys and 192 girls) between 8 and 10 years of age (*M*_*age*_ = 9.2, *SD* = 0.4). Participants were pupils of state elementary schools of the Capital City of Prague, Czech Republic, and were selected using an intentional selection process. More specifically, participants were selected from schools without art, sport, language or technical specializations. Furthermore, children could not be enrolled in “integrated classes” for children with special needs. Beyond the aforementioned inclusion criteria, other factors which may influence performance (e.g., activities outside of school, including sports, hobbies and activities) were not considered. This was a limitation.

Participants were selected using the following purposefully method of sampling. In cooperation with the Institute of Educational and Psychological Counselling, a complete list of primary schools from each district in the City of Prague was obtained. Only those schools that were attended by at least 50 individuals of the given age were selected, the number of the participants per school was set at 40. Out of these schools, a list was created from which one primary school was randomly selected from each district of Prague. In total, 10 primary schools were selected. The Ethics commission of the Faculty of Physical Education and Sport, at Charles University granted ethics approval. Written parental consent was obtained for all children.

### Apparatus and Procedures

#### Hand preference tasks

To determine hand preference, five unimanual motor tasks were implemented [51]. Assessments have been used in previous studies [13, 19, 52], and have been validated for use with Czech children [51]. The tasks included: (1) draw a leaf according to the model **(Draw)**; (2) take the bell in one hand and ring it **(Ring)**; (3) take the ball in one hand and throw it at the target (three attempts; **Throw**); (4) show how many points you can roll with the dice (three attempts; **Cube**); and (5) demonstrate how you brush your teeth **(Brush)**. Tasks were scored dichotomously, where 0 indicated the task was performed with the left hand and 1 indicated performance with the right hand. Throw, Cube, Cards and Matches tasks included repetition; therefore, scores comprised of a sum of attempts performed with the right hand.

#### Bishop’s card reaching task (Bishop’s)

Participants were seated on a chair at a desk for the duration of the study. The researcher placed a sheet of paper (42 cm x 29.7 cm; divided in half by a vertical line) on the desk in front of the participant. The paper contained seven rectangular boxes (6 cm × 3 cm) at successive 30 degree intervals forming a semicircle. There were three boxes in left space, one at the midline, and three boxes in right space. Each box was labelled from -3 (far left) to +3 (far right), with the box at the midline labelled 0. The researcher placed a card (all different colors) in each box and asked the participant to turn the card of a designated color using one hand. The card placed at the midline was selected first. If the participant used the right hand, selection progressed in the following order: +2, –2, +3, –3. If the participant used the left hand, selection progressed in the following order: –2, +2, –3, +3. After each trial (i.e., after each card was selected) hand selection was recorded on a score sheet. A value of 0 indicated left hand selection; whereas, a value of 1 indicated right hand selection.

### Data Analysis

To analyse the relationship between manifest indicators and latent variables with continuous characters, structural equation modelling (SEM) is recommended [45, 53]. Therefore, SEM was used to assess the relationship between data obtained from the hand preference tasks and Bishop’s card reaching task. One SEM approach includes factor analysis, which has been used in previous studies to determine the structure or diagnostic quality of handedness questionnaires [54, 55, 56]. In this study, hand preference was defined as a latent continuous variable with dichotomous (or ordered categorical type) scoring; therefore, categorical confirmatory factor analysis (CCFA) was selected as an appropriate psychometric approach from SEM. This method is sometimes called IRT [57] or item factor analysis (IFA) [58]. It is suitable to test or verify structural theories, and to test the validity of a certain tool [48, 58, 59]. The weighted least-squares (WLSMV) approach was selected as the estimation parameter as recommended in Muthén [60].

The quality of structural models was evaluated according recommended cut-off lines using five fit indices: (1) Chi-square test (Sattora-Bentler scaled chi-square); (2) Comparative fit index (CFI) [61], >.95; (3) Root mean square of approximation (RMSEA) [62], < .06; (4) Tucker-Lewis index (TLI) [63], >.95; and (5) Weighted Root Mean Square Residual (WRMR)[60], < .80. Sensitivity of the QHP Bishop’s card reaching task was evaluated using chi-square contingency tables. Data were analyzed in M-plus 6 [64]and NCSS2007 (NCSS, LLC, Kaysville, UT).

## Results and Discussion

Six indicators evaluating the latent variable “hand preference” were modeled using CCFA. The proposed uni-dimensional model displayed very good values of fit and high factor loading of all six indicators of hand preference (see Table 1). In this one-factor structure, Bishop’s card reaching task proved to be a suitable indicator of hand preference (factor validity = .89; see Table 2); however, high factor loads revealed a possible multicollinearity of some motor tasks (i.e., excessive mutual correlation). A correlation matrix was created to verify the discriminant and convergent validity of individual indicators. Polychoric correlations were used for ordered categorical data, and tetrachoric correlations were used for dichotomously (binary) scored data.

**Table 1:** Fit of the one-factor model.

Model	Chi-square	P-value	df	CFI	TLI	RMSEA	WRMR
1-factor	21.58	.08	9	.97	.97	.051	.522

**Table 2:** One-factor model.

	Six items	
Items	Factor Loadings	Uniqueness
Draw a leaf according to the model–**Draw**	.96	.08
Take the bell in one hand and ring it–**Ring**	.90	.19
Take the ball in one hand and throw it at the target–**Throw**	.92	.15
Show how many points you can roll with the dice on three attempts–**Cube**	.98	.04
Demonstrate how you brush your teeth–**Brush**	.94	.12
Bishops’ card reading task–**Bishop’s**	.89	.21
Cronbach’s α	.89	

The correlation matrix clearly displays a strong (>.90) correlation between the individual indicators (see Table 3). On average, the weakest correlations (though still at high levels) were in Bishop’s card reaching task. Detailed analysis showed that two motor tasks, **Brush** and **Draw**, have the strongest correlations with other tasks. Based on the analysis of motor activity and with regard to possible sociocultural pressures on hand preference in these two tasks, we decided to exclude **Brush** and **Draw** items and thus verify their redundancy in the proposed one-factor model (see Table 4).

**Table 3:** Correlation matric of six motor tasks (including Bishop’s card reaching task).

Items	Brush	Throw	Ring	Draw	Bishop’s	Cube
Brush						
Throw	.988					
Ring	.956	.968				
Draw	.985	.983	.957			
Bishop’s	.921	.896	.859	.868		
Cube	.959	.918	.897	.918	.864	

**Table 4:** Fit of the one-factor model without Brush and Draw tasks.

Model	Chi-square	P-value	df	CFI	TLI	RMSEA	WRMR
1-factor	6.58	.25	2	.99	.99	.039	.351

After excluding the Brush and Draw motor tasks, the one-factor model with four indicators showed a significant improvement in fit values (see Table 5). The most marked proved to be changes in significance of the model (*p* = .25) and chi-square, whose value was considerably lower statistically, at the level *p* < .001, even with the relevant number of degrees of discretion. This improvement in fit in spite of the model restriction (which means a certain loss of information) indicated that the **Brush** and **Draw** tasks were highly redundant in the model.

**Table 5:** One factor model without Brush and Draw tasks.

	Six Items		Four Items	
Items	Factor Loadings	Uniqueness	Factor Loadings	Uniqueness
Draw a leaf according to the model–**Draw**	.96	.08	-	-
Take the bell in one hand and ring it–**Ring**	.90	.019	.91	.17
Take the ball in one hand and throw it at the target–**Throw**	.92	.15	.92	.15
Show how many points you can roll with the dice on three attempts–**Cube**	.94	.12	.93	.13
Demonstrate how you brush your teeth–**Brush**	.98	.04	-	-
Bishop’s card reaching task–**Bishop’s**	.89	.21	.89	.21
Cronbach’s α	.89		.84	

To evaluate sensitivity of Bishop’s card reaching task, participants were divided into two groups based on an absolute preference for the right (*n* = 308) and left (*n* = 31) hand. This division was based on performance of the five hand preference tasks previously standardized for use with Czech children [50]: Draw, Brush, Cube, Ring and Throw. Participants who performed all tasks with the right hand were described as having an absolute preference for the right hand; whereas, those who performed all tasks with the left hand were described as having an absolute preference for the left hand. Chi-square tests with contingency tables revealed the Bishop’s card reaching task had sufficient sensitivity for identifying absolute right-handers (*n* = 306) and left-handers (*n* = 31; see Table 6). In addition, these results showed that the sensitivity of Bishop’s card reaching task is approximately 7% lower (right-handers = 97.4% and left-handers = 90.3%) in identifying left- handers compared to right-handers. Nevertheless, chi-square criterion showed that this difference was not significant.

**Table 6:** Sensitivity of Bishop’s card reaching task according to the instructions “turn the cards of the given colour placed on the sheet of paper.”

How the task was proved	Right handers	Left handers
All cards were taken by right hand	298	0
All cards were taken by left hand	0	28
At least one card was taken by non-preferred hand	8	3
Total	306	31

The current study aimed to assess the factor validity of Bishop’s card reaching task. Furthermore, to examine discriminant and convergent validity between Bishop’s card reaching task and a selection of five standard assessments of hand preference in Czech children. Structural equation modeling was used to determine the diagnostic value of Bishop’s card reaching task, along with select unimanual indicators, with the defined latent variable “hand preference.” Confirming our hypothesis, the factor validity of Bishop’s card reaching task indicated it was a suitable indicator of hand preference (.89), and successfully identified hand preference in children. More specifically, assessments of sensitivity revealed identification of 90.3% of left-handers and 97.4% of right-handers. It is important to note that, although sensitivity levels were 7% greater in right-handers, the difference was non-significant. This finding supports previous results that left-handers represent a more heterogeneous population in terms of cerebral lateralization for handedness [65, 66].

Findings of the current study are in agreement with previous reports. In the original study, Bishop et al. [24] used the card-reaching task to distinguish between right-handed young adults based on degree of hand preference. Carlier et al. [39] has used Annett’s Questionnaire and Bishop’s card reaching task to assess the hand preference of 3- to 10-year-olds (right- and left-handed); however, did not assess the correlation between the two measures. Nevertheless, regardless of the method used to classify hand preference, analysis of the number of midline crossings remained the same for both handedness groups. Using the same methods, Doyen et al. [40] identified a significant, yet low (.23) correlation between measures with right-handed individuals ages 6 to 66, in line with findings from the current study. Likewise, Hill and Khanem [41] reported, “a child’s age and peg-moving speed had a significant influence on the likelihood of using their preferred hand to point and reach” (p. 105). The Edinburgh Handedness Questionnaire [19] and an unspecified peg-moving task were used in their study with 4- to 11-year-olds [41]. Taken in light of the current findings, where greater than 90% of participants (90.3% of left-handers and 97.4% of right-handers) were successfully identified according to their hand preference, it can be argued that using Bishop’s card reaching task is a suitable and valid method to measure handedness.

That said, although the aforementioned work used Bishop’s card reaching task to distinguish between handedness groups, it is important to highlight discrepant findings in the literature as well. In a follow-up to Bishop et al. [24], Calvert and Bishop [33] added two tasks (point, place) to extend the QHP, and assess the relationship with Annett’s peg moving task as a traditional assessment. All QHP tasks were significantly correlated with Annett’s peg moving task; however, only point and placing were able to separate strong and weak right- and left-handers. For card reaching, there was an evident trend in this direction, where the task was able to differentiate between left-handers based on degree of hand preference, but not right-handers. Doyen and Carlier [29] were also unable to replicate Bishop et al.’s [24] findings. Although homogeneity and test-retest reliability were revealed, the ability to sort participants into subgroups of hand preference was not achieved by Bishop’s card reaching test, in particular with left-handers. Doyen and Carlier [29] thus suggested that both preference and performance tasks should be implemented to ensure a complete assessment of handedness, considering differences in cerebral lateralization between right and left handers. Nevertheless, it was also acknowledged that differences are likely attributed to the way in which handedness groups are classified, and the tasks used to divide participants based on degree of handedness [29].

As evidenced in the current study, tasks must be carefully considered when assessing hand preference. Findings revealed that the one factor model which included all five unimanal tasks (see Table 1) showed acceptable fit values (P = .08, RMSEA .051, CFA .97, WRMR .522); however, psychometric problems with strong multicollinearity were also revealed. A correlation matrix was thus created in order to determine the relationship between individual motor tasks in order to identify any potential redundancies. Strong correlations emerged for Brush and Draw tasks (>.90; see Table 3), similar to Komarc and Harbichová [67] who also noted possible redundancy of some motor tasks in their model. These tasks (i.e., Brush and Draw) were thus excluded from further analysis, even though the restriction of the model means a certain loss of information [58]. Despite both of these tasks recognized as most relevant in the handedness literature [11, 19], removing these tasks significantly improved the fit of the unidimensional model (see Table 4). More specifically, chi-square values decreased and the model significance increased (P = .25).

On the basis of these findings, it can be argued that Draw and Brush motor tasks are likely more influenced by sociocultural pressures than other tasks, and consequently were redundant in the model. Cultural influences have been shown to play a role in the development of handedness. In particular, western cultures typically show a higher incidence of left handedness than eastern cultures. For example, comparisons of hand preference in India and North America [68], and Japan and Canada [69] have noted the number of left-handers is considerably lower due to social constraints limiting left hand use [69]. Relevant to the current study, sociocultural pressures have been reported to have primary effects on skilled activities such as writing and eating [69]. As such, it is likely that Draw and Brush tasks may not accurately reflect handedness and cerebral lateralization in everyday settings. Furthermore, these differences may explain why the sensitivity for left-handers was 7% lower than right-handers.

## Conclusions

Taken together, findings from the current study revealed that Bishop’s card reaching task displayed a significant relationship with the latent variable “hand preference” and previously verified unimanual tasks. Moreover, Bishop’s card reaching task was found to have high sensitivity in discriminating between right-handers and left-handers. That said, it is important to notice that Bishop’s card reaching task expressed higher sensitivity for right-handers in comparison to left-handers. In summary, these findings support the idea that Bishop’s card reaching task is a suitable and valid approach to assess hand preference in 8- to 10-year-old children. Notwithstanding the previous, it is important to acknowledge that the current study assess hand preference in a relatively homogenous narrow age range (i.e., 8 to 10), stereotypically known to display consistent hand preference, indicative of strong cerebral lateralization. Furthermore, participants were recruited using purposeful sampling. It is also important to acknowledge that the unimanual tasks used in the current study was not a fully exhaustive battery; however, do reflect the most commonly used assessments of hand preference in the literature. Future research should thus aim to replicate findings in a broader age range of children, to provide a foundation for examining neurodevelopmental disorders (e.g., Autism Spectrum Disorders, Attention Deficit Hyperactivity Disorders, etc.), where the proportion of right- and left-handers is disrupted in comparison to typically-developing peers. For example, neural deficits characteristics of Autism Spectrum Disorders are stereotypically of left hemisphere functions; therefore, a link between non-right-handedness, learning disability and left hemisphere dysfunction has become prevalent in the literature [10, 70]. In another example, Hill and Bishop [71] used the QHP to compare 7- to 11-year-olds with specific language impairment and developmental coordination disorder to their age-matched peers and younger control group. Findings revealed the ability to be a more sensitive, albeit not specific, indicator of developmental disorder than a traditional handedness questionnaire (i.e., Edinburgh Handedness Questionnaire) [19].
